# Highly conserved salt bridge stabilizes a proteinase K subfamily enzyme, Aqualysin I, from *Thermus aquaticus* YT-1

**DOI:** 10.1186/s13568-014-0059-2

**Published:** 2014-08-13

**Authors:** Masayoshi Sakaguchi, Kanae Osaku, Susumu Maejima, Nao Ohno, Yasusato Sugahara, Fumitaka Oyama, Masao Kawakita

**Affiliations:** 1 Department of Applied Chemistry, Kogakuin University, 2,665-1 Nakano-cho, Hachioji 192-0015, Tokyo, Japan; 2 Center for Medical Research Cooperation, Tokyo Metropolitan Institute of Medical Science, 2-1-6 Kami-kitazawa, Setagaya-ku 156-8506, Tokyo, Japan

**Keywords:** Serine protease, Subtilase, Proteinase K subfamily, Salt bridge, Thermal stability

## Abstract

The proteinase K subfamily enzymes, thermophilic Aqualysin I (AQN) from *Thermus aquaticus* YT-1 and psychrophilic serine protease (VPR) from *Vibrio* sp. PA-44, have six and seven salt bridges, respectively. To understand the possible significance of salt bridges in the thermal stability of AQN, we prepared mutant proteins in which amino acid residues participating in salt bridges common to proteinase K subfamily members and intrinsic to AQN were replaced to disrupt the bridges one at a time. Disruption of a salt bridge common to proteinase K subfamily enzymes in the D183N mutant resulted in a significant reduction in thermal stability, and a massive change in the content of the secondary structure was observed, even at 70°C, in the circular dichroism (CD) analysis. These results indicate that the common salt bridge Asp183-Arg12 is important in maintaining the conformation of proteinase K subfamily enzymes and suggest the importance of proximity between the regions around Asp183 and the N-terminal region around Arg12. Of the three mutants that lack an AQN intrinsic salt bridge, D212N was more prone to unfolding at 80°C than the wild-type enzyme. Similarly, D17N and E237Q were less thermostable than the wild-type enzyme, although this may be partially due to increased autolysis. The AQN intrinsic salt bridges appear to confer additional thermal stability to this enzyme. These findings will further our understanding of the factors involved in stabilizing protein structure.

## Introduction

The molecular bases of protein adaptation to high and low temperatures are interesting from both basic and practical standpoints, as knowledge regarding these factors would enable the construction of genetically engineered proteins that could function under a variety of conditions. Psychrophilic and mesophilic enzymes are used in biotechnological applications requiring high activity at mild temperatures or quick heat-inactivation at moderate temperatures. Thermophilic and hyperthermophilic enzymes have major biotechnological advantages over mesophilic and psychrophilic enzymes because of their high activities at higher temperatures and substrate concentrations as well as their resistance to chemical denaturants. Thus far, various intramolecular interactions, including ionic interactions, hydrogen bonding and hydrophobic interactions, are assumed to make important contributions to the stability and maintenance of enzyme structure as well as the catalytic functions; however, their contributions have not been fully defined in individual cases. In addition, the comparative structural analysis of psychrophilic, mesophilic and thermophilic enzymes indicated that each protein family adopts a different structural strategy to adapt to different temperature ranges (Siezen & Leunissen [1997]; Struvay & Feller [2012]). To understand the thermal adaptation strategy of proteins, comparative studies among members of a protein family using site-directed mutagenesis as well as laboratory evolution via random mutagenesis using error-prone PCR will provide valuable information.

According to the MEROPS peptidase database (http://merops.sanger.ac.uk/), subtilisin-like protease (subtilase) superfamily members are classified as the S8 subfamily in the serine protease superfamily. These proteins exhibit a highly conserved arrangement of amino acids in the active site and have very similar overall structures consisting of an α/β protein scaffold. Nevertheless, their temperature stability profiles differ widely, and they may be psychrophilic, mesophilic, thermophilic or hyperthermophilic depending on the characteristics of the organisms from which they are derived. Because of these characteristics, they appear to be suitable for comparative studies to elucidate the basis of structure-function relationships. Aqualysin I (AQN) is an alkaline serine-protease produced by the Gram-negative thermophilic bacterium *Thermus aquaticus* YT-1 (Matsuzawa et al. [1983]; Matsuzawa et al. [1988]). Based on an analysis of sequence homology, AQN is classified into the proteinase K subfamily, which consists of a group of Gram-negative bacteria-derived proteinases within the subtilase superfamily (Siezen & Leunissen [1997]). In our previous study, we demonstrated that Pro residues in the surface loops of AQN in the N-terminal region contribute significantly to its thermophilicity, and one of two disulfide bonds in AQN is more important for the catalytic activity and conformational stability of AQN than the other (Sakaguchi et al. [2007]; Sakaguchi et al. [2008b]). These results are consistent with those reported for a subtilisin-like serine protease from *Vibrio* sp., VPR, which is a psychrophilic counterpart of AQN in the proteinase K subfamily (Kristjánsson et al. [1999]; Arnórsdóttir et al. [2002]). It was found that the introduction of Pro residues into VPR at positions corresponding to those in AQN could improve its thermal stability (Arnórsdóttir et al. [2009]).

To enhance the thermal stability of a protein, a common strategy is to introduce more favorable surface charge-charge interactions. However, the role of salt bridges in the stabilization of proteins remains controversial. Thermophilic proteins have an increased number of salt bridges compared with their mesophilic homologues (Kumar et al. [2000]; Szilágyi & Závodszky [2000]; Vogt et al. [1997]). In VPR, a psychrophilic enzyme, seven salt bridges (Arg10-Asp183, Arg14-Asp274, Asp56-Arg95, Asp59-Arg95, Asp138-Arg169, Arg185-Asp260 and Glu236-Arg252) have been identified in the known structure of the enzyme (PDB accession number: 1SH7; (Arnórsdóttir et al. [2005])). However, AQN has six salt bridges (Arg12-Asp183, Asp17-Arg259, Arg31-Asp237, Arg43-Asp212, Asp58-Arg95 and Asp138-Arg169) in its structure (4DZT; (Green et al. [1993])) (Table 1). Both enzymes have a similar number of salt bridges; however, their thermal stabilities are quite different. The VPR N15D mutant, in which an Asp residue is substituted for Asn15 to form a new salt bridge (Asp15-Lys257) at the position corresponding to the Asp17-Arg259 salt bridge in AQN, exhibited increased thermal stability due to the incorporation of a new salt bridge; the thermal stability of the enzyme did not increase further in the double mutant VPR N15D/K257R (Sigurdardóttir et al. [2009]). Inversely, the deletion of the Asp17-Arg259 salt bridge in the AQN D17N mutant resulted in reduced thermal stability compared to the wild-type enzyme without exerting a significant effect on the kinetic parameters of the hydrolysis reaction (Arnórsdóttir et al. [2011]). These results may imply that salt bridges at appropriate locations play a vital role in the thermal stability of serine proteases, particularly those of the proteinase K subfamily. To help clarify this complicated issue and to further extend our understanding of the molecular basis of proteinase K-related enzyme stabilization, we aimed to examine the role of salt bridges in AQN by site-directed mutagenesis.

**Table 1:** Amino acids that form salt bridges in AQN, VPR and SPRK

	**AQN (4DZT)**	**VPR (1SH7)**	**SPRK (2B6N)**
Common	Arg12-Asp183	Arg10-Asp183	Arg12-Asp187
	Asp58-Arg95	Asp56-Arg95	Asp58-Arg97
	Asp138-Arg169	Asp138-Arg169	Asp140-Arg171
Intrinsic to VPR or SPRK		Asp59-Arg95	Asp61-Arg97
		Arg185-Asp260	Arg187-Asp264
		Glu236-Arg252	Asp240-Arg256
		Arg14-Asp274	Asp2-His25
			Asp198-Lys254
Intrinsic to AQN	Asp17-Arg259		
	Arg31-Glu237		
	Arg43-Asp212		

## Materials and methods

### Strains and growth medium

*E. coli* TG1 was used as the expression host, and *E. coli* DH5α (TOYOBO, Osaka, Japan) and MV1184 (TAKARA BIO INC., Shiga, Japan) were used as the genetic engineering hosts. LB medium (1% Bacto-tryptone, 0.5% Bacto-yeast extract, 1% NaCl, pH 7.0) was used. The solid medium contained Bacto-agar (1.5%). Ampicillin (50 μg/ml) or kanamycin (50 μg/ml) was added to the medium as needed.

### Genetic engineering and chemical reagents

Genetic engineering experiments were performed according to the procedure described by Sambrook and Russell (Sambrook & Russell [2012]). The enzymes used for genetic engineering were purchased from TAKARA BIO and used according to the manufacturer’s instructions. Bacto-tryptone and Bacto-yeast extract were purchased from Becton Dickinson (Franklin Lakes, NJ, USA). Other reagents used were of the highest quality available and were obtained from Wako Pure Chemicals (Tokyo, Japan) and Sigma-Aldrich (St. Louis, MO, USA), unless otherwise specified.

### Plasmid construction

The plasmid pMAQΔc2, which was designed to express wild-type AQN as a fusion protein with maltose binding protein (MBP), was constructed based on pAQNΔC105 and pMAL plasmids (New England Biolabs, Ipswich, MA, USA) as described previously (Sakaguchi et al. [2008a]).

To construct plasmids with a mutated aqualysin I gene, site-directed mutagenesis was performed following the ODA-PCR method (Mutan®-Super express Km; TAKARA BIO) using pMAQΔc2 as a template. The oligonucleotide primers (Sigma-Aldrich Life Science, Hokkaido, Japan) used for site-directed mutagenesis are shown in Table 2. The fragments containing either of the mutations listed in the table were inserted into the expression vector, pMAQΔc2. The names of the mutant plasmids are provided in the third column of Table 2 (D17N, *etc*.). The nucleotide sequences around the mutation sites as well as other parts of the gene were confirmed by DNA sequencing using an Applied Biosystems 3130 Genetic Analyzer (Applied Biosystems, Foster City, CA, USA).

**Table 2:** Oligonucleotide primers used for site-directed mutagenesis

**Name**	**Oligonucleotide sequence**	**Mutant name**
AQN-D17N	5*'*-CCAGCGG*A*ACCTTCCC-3*'*	D17N
AQN-D58N	5*'*-GGTAGGCTAT*A*ACGCCTTAGGG-3*'*	D58N
AQN-G61D	5*'*-ACGCCTTAG*AT*GGGAACG-3*'*	G61D
AQN-D138N	5*'*-CTGCCCTG*A*ACAACGCC-3*'*	D138N
AQN-D183N	5*'*-ATCTTCC*A*ACGCCCGTG-3*'*	D183N
AQN-D212N	5*'*-ACACCTCG*A*ACACGGCC-3*'*	D212N
AQN-E237Q	5*'*-CTTTGTATCTA*C*AGCAAAATCTTC-3*'*	E237Q
AQN-G262D	5*'*-GCTTTCGG*A*TATCGGATCG-3*'*	G262D
AQN-S277D	5*'*-CCTGCTC*GAT*TCGGGGAG-3*'*	S277D

Mismatched bases are shown in italic.

### Purification and activity measurement of the wild-type enzyme and its mutants

After induction by isopropyl β-D-thiogalactopyranoside (IPTG) at OD_660_ = 0.8, the transformants were further cultivated overnight in LB medium. The cells were harvested by centrifugation and subsequently sonicated, and the crude extract was subjected to heat treatment, hydrophobic chromatography (Butyl Sepharose; GE Healthcare, Buckinghamshire, UK) and cation exchange chromatography (Resource S; GE Healthcare) as described previously (Sakaguchi et al. [2008a]). The enzymes were purified to homogeneity to yield a single band on SDS-polyacrylamide gel electrophoresis (PAGE) after staining with Coomassie Brilliant Blue R-250 (CBB R-250) (Laemmli [1970]). Prior to SDS-PAGE analysis, the enzymes were treated with 25 mM phenylmethane sulfonyl fluoride (PMSF) dissolved in methanol for 30 min to prevent autolytic degradation. To minimize denaturation and autolysis which may occur at higher temperature, the enzyme activity was measured at 40°C with *N*-succinyl-Ala-Ala-Pro-Phe-*p*-nitroanilide (*N*-suc-AAPF-pNA, Sigma-Aldrich) as a substrate in 50 mM 2-[4-(2-hydroxyethyl)-1-piperazynyl]ethanesulfonic acid (HEPES)-NaOH (pH 7.5) buffer containing 1 mM CaCl_2_. The change in absorbance at 410 nm was continuously monitored, and the activity was estimated using ε_410_ = 8,680 M^−1^ cm^−1^ as a molar absorption coefficient of *p*-nitroaniline (4-nitroaniline). One unit of enzyme was defined as the amount of enzyme that liberates 1 μmole of *p*-nitroaniline from the substrate in 1 minute. The protein concentration was measured using the micro-assay method (Bio-Rad Laboratories, Hercules, CA, USA), which is based on the Bradford method (Bradford [1976]), using bovine serum albumin as a standard.

### Determination of the temperature dependence of the proteolytic activity and heat stability of the wild-type enzyme and its mutants

To examine the temperature dependence of the enzyme activity, 480 μl of 1 mM *N*-suc-AAPF-pNA solution in 100 mM HEPES-NaOH (pH 7.5) containing 1 mM CaCl_2_ was preincubated at an appropriate temperature for 5 min; subsequently, 20 μl of enzyme solution (5 μg/ml) was added. The change in absorbance at 410 nm was continuously monitored, and the activity was estimated as described above based on the results of triplicate experiments.

To examine the heat stability, the enzymes were diluted with 20 mM 2-morpholinoethanesulfonic acid (MES)-NaOH buffer (pH 6.0) containing 1 mM CaCl_2_ to yield a 10 μg/ml solution. These experiments were performed at pH 6.0 to diminish the massive autolysis that would occur under more alkaline condition. This enabled us to observe the differential decrease of residual activity among mutants due to structural destabilization during the heat treatment processes. The enzyme solution was incubated for the appropriate time period at 70°C or 80°C and was subsequently cooled quickly. The remaining activity was determined based on the results of triplicate experiments using 1 mM *N*-suc-AAPF-pNA as a substrate at 40°C, as described above.

### Kinetic analysis

The initial rates of *N*-suc-AAPF-pNA hydrolysis induced by the wild-type enzyme and mutant enzymes were measured at 40°C in 50 mM HEPES-NaOH (pH 7.5) containing 1 mM CaCl_2_ as described above. The kinetic parameters *V*_max_ and *K*_m_ were estimated assuming a Michaelis-Menten kinetic model, and the graphics software package DeltaGraph version 6 (Nihon Poladigital K.K., Tokyo, Japan) was used with non-linear regression. The apparent values of *k*_cat_ were estimated using a molecular mass of 28 kDa.

### Unfolding study of AQN proteins based on circular dichroism (CD) measurement

CD analysis was carried out to determine the transition temperature (*T*_m_) and to monitor the unfolding of the wild-type and mutant enzymes. Prior to the CD measurements, purified enzymes were treated with 25 mM PMSF dissolved in methanol for 30 min to prevent autolytic degradation during the measurements. After complete inactivation of the protease activity was confirmed, the samples were dialyzed overnight against 20 mM MES-NaOH buffer (pH 6.0) containing 1 mM CaCl_2_ and filtered through a MILEX®-HV filter (0.45 μm pore size, Durapore (PVDF), Merck Millipore Ltd., Carrigtwohill, Ireland). CD measurements were conducted using a JASCO-725 circular dichroism spectropolarimeter equipped with PTC-348 Peltier type single cell holder, and the change in ellipticity at 220 nm was monitored under a constant heating rate (1°C/min) at temperatures ranging from 40 to 105°C. The melting curves were normalized according to the methods in the literature (Arnórsdóttir et al. [2002]), and the melting temperature (*T*_m_) values of the enzymes were estimated using a graphics software package, Delta graph. The experiments were performed in duplicate.

The unfolding of proteins as a function of time was observed at a constant temperature (70°C or 80°C) by CD measurement over a range of 200–250 nm. Measurements were performed with a JASCO-725 circular dichroism spectropolarimeter (JASCO, Tokyo, Japan) equipped with PTC-348 Peltier type single cell holder, and the change in ellipticity was monitored at every 5 min over a 30-min period at a constant temperature. Wavelength scans in the range of 200–250 nm were performed in rectangular quartz cells (JASCO model: T-11-ES-1) with a path length of 0.1 cm.

## Results

### Mutagenesis of salt bridge-forming residues in AQN and purification of the wild-type enzyme and its mutants

**Figure 1:**
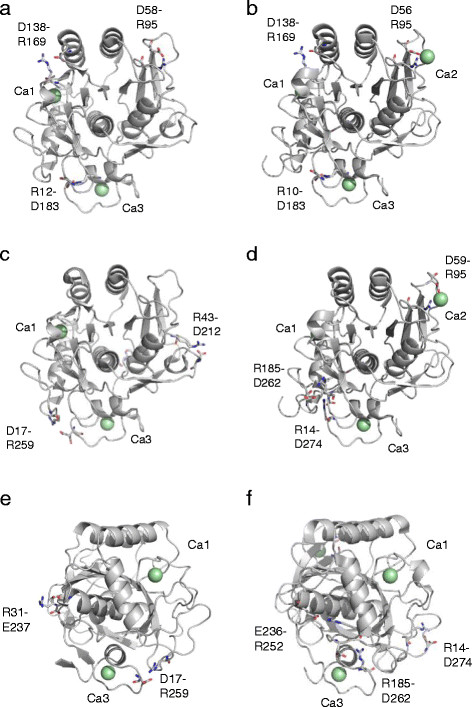
**Crystal structures of AQN (a, c, e; 4DZT) and VPR (b, d, f; 1SH7). (a, b)** Common salt bridge positions, **(c, d)** intrinsic salt bridge positions, **(e, f)** intrinsic salt bridge positions rotated approximately 90 degrees horizontally from **(c, d)**. The salt bridges of interest are indicated by residue numbers and are represented as stick models. Calcium ions (Ca1, Ca2 and Ca3) are represented as pale green spheres.

Table 1 lists the salt bridge-forming amino acid residues in AQN, VPR and *Serratia* proteinase K-like enzyme (SPRK; (Larsen et al. [2006])), which were identified based on the known structures of AQN (4DZT; (Green et al. [1993])), VPR (1SH7; (Arnórsdóttir et al. [2005])) and SPRK (2B6N; (Helland et al. [2006])), respectively. There are three conserved salt bridges in both AQN and VPR (Figure 1a and b). In addition, AQN and VPR have three (Asp17-Arg259, Arg31-Glu237 and Arg43-Asp212) and four (Asp59-Arg95, Arg14-Asp274, Arg185-Asp260 and Glu236-Arg252) intrinsic salt bridges, respectively (Figure 1c, d, e and f). To examine the role of salt bridges in AQN, the Asp and Glu residues were replaced with Asn and Gln residues, respectively, to make them incapable of forming salt bridges. To introduce new salt bridges in AQN at the positions at which the VPR-intrinsic salt bridges are located, the residues Gly61, Gly262 and Ser277 were converted to Asp residues to make them capable of forming salt bridges with Arg95, Arg185 and Arg16, respectively, which are conserved in both AQN and VPR. The mutant AQN constructs were expressed, and the protein products were purified to homogeneity using essentially the same methodology used for the wild-type enzyme.

### Thermal stability of the wild-type enzyme and its mutants

**Figure 2:**
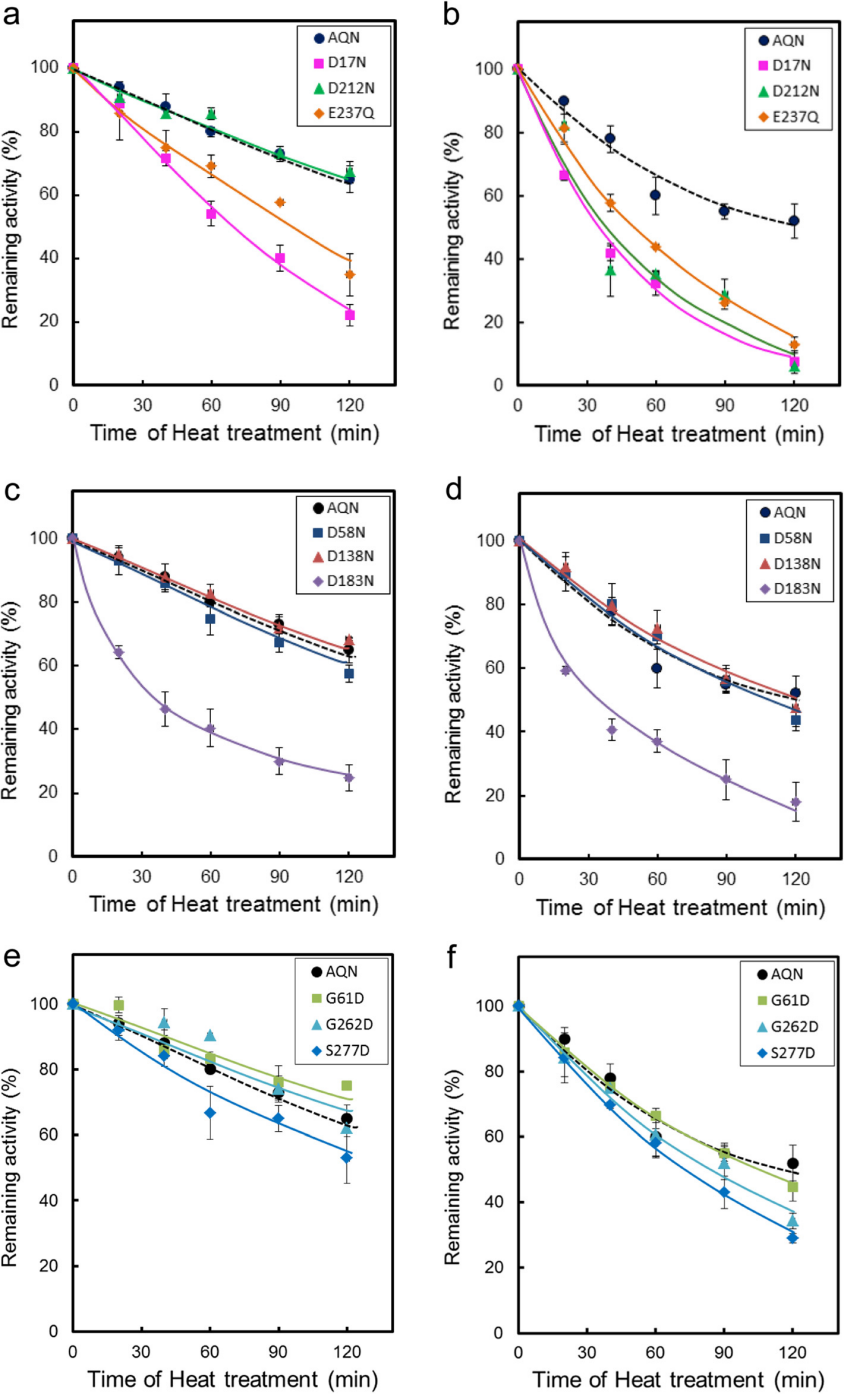
**Heat stability of the wild-type and mutant enzymes.** Heat stability was determined as described in the Materials and methods section. The remaining activities after heat treatment at 70°C **(a,c and e)** or 80°C **(b,dandf)** for the indicated times relative to the activity before heat treatment are plotted for each mutant. **(a, b)** mutants lacking an intrinsic salt bridge, **(c, d)** mutants lacking a common salt bridge, **(e, f)** mutants in which a salt bridge may have been introduced.

The remaining activity of the wild-type and mutant enzymes after heat treatment at temperatures of 70°C or 80°C was determined at 40°C using *N*-suc-AAPF-pNA as a substrate; the results are illustrated in Figure 2a-f. Figure 2a and b show the time courses of the residual activity at 70°C and 80°C, respectively, for the D17N, D212N and E237Q mutants lacking a salt bridge specific to AQN. At 70°C, the activity of E237Q and D17N decreased more rapidly than that of the wild-type enzyme, indicating that the mutants are less stable at 70°C. D212N behaved similarly to the wild-type enzyme at 70°C; however, at 80°C, it was inactivated as rapidly as E237Q and D17N (Figure 2a and b). These results indicate that the AQN-intrinsic salt bridges Arg31-Glu237, Asp17-Arg259 and Arg43-Asp212 contribute significantly to the maintenance of active enzyme structures at temperatures above 70-80°C.

Disruption of the salt bridges common to AQN and VPR did not yield a common outcome. For example, the residual activity of D183N declined rapidly, with a half-life of approximately 30 min at both 70°C and 80°C, whereas the activities of D58N and D138N were almost indistinguishable from that of the wild-type enzyme at both temperatures (Figure 2c and d). The above results indicate that the Arg12-Asp183 salt bridge is important for conferring structural stability to proteinase K subfamily enzymes, although the other two common salt bridges are not significantly involved in thermal stabilization. Additional salt bridges were introduced at the positions where VPR-intrinsic salt bridges are located in mutants G61D, G262D and S277D. The inactivation time courses of the three mutants were similar to that of the wild-type enzyme at 70°C and 80°C. In fact, S277D was slightly less stable than the wild-type enzyme at both 70°C and 80°C (Figure 2e and f).

### Temperature dependence of wild-type and mutant enzyme activity

**Figure 3:**
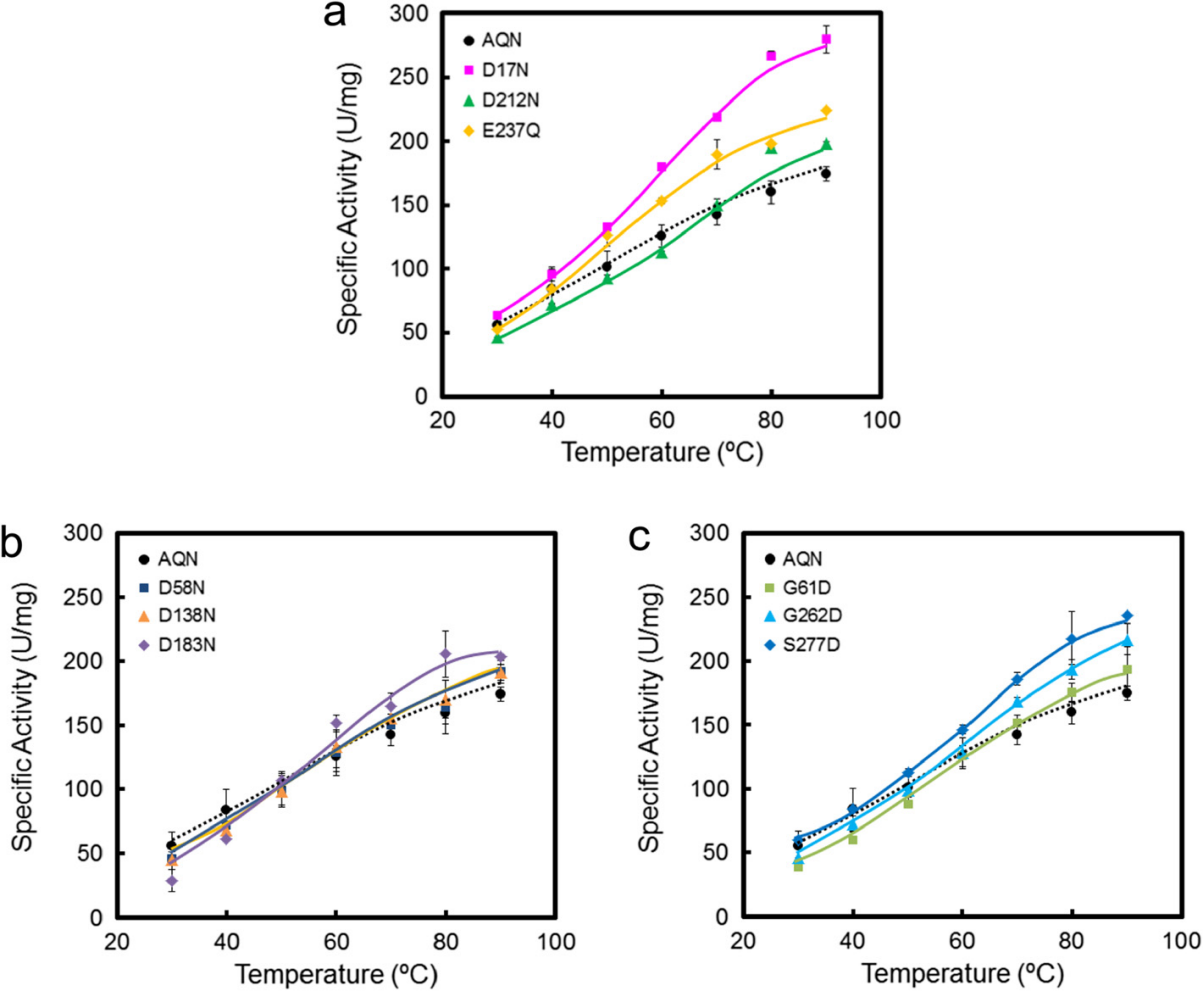
**Temperature-dependence of the activity of the wild-type and mutant enzymes.** The temperature-dependence of the activity was measured as described in the Materials and methods section. **(a)** mutants lacking an intrinsic salt bridge, **(b)** mutants lacking a common salt bridge, **(c)** mutants in which a salt bridge may have been introduced.

Figures 3a-c compares the temperature dependence of wild-type and mutant enzyme activity. All mutants displayed similar temperature-activity profiles over a range of 30-90°C. Disrupting a salt bridge or introducing a potential salt bridge-forming mutation did not lead to an extensive reduction of enzyme activity. In fact, the enzyme activity of the mutants tended to be slightly higher than that of the wild-type enzyme especially at higher temperatures. D17N showed the highest activity among the mutants tested: its activity was about 1.5-fold as high as that of the wild-type enzyme at 90°C. The optimum temperature was not altered significantly by the mutations, and every mutant showed full activity at approximately 90°C, similar to the wild-type enzyme.

### Kinetic analysis of wild-type and mutant enzymes

**Figure 4:**
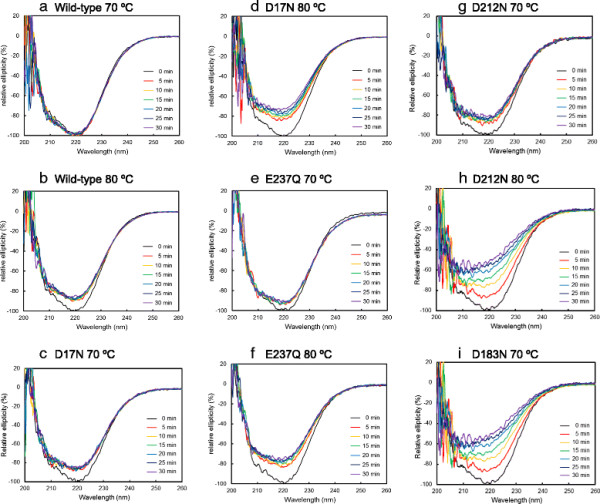
**The change in CD spectra of the wild-type and mutant enzymes over a range of 0–30 min at a constant temperature. (a)** Wild-type enzyme at 70°C, **(b)** wild-type enzyme at 80°C, **(c)** D17N at 70°C, **(d)** D17N at 80°C, **(e)** E237Q at 70°C, **(f)** E237Q at 80°C, **(g)**, D212N at 70°C, **(h)**, D212N at 80°C, **(i)** D183N at 70°C. The experimental conditions are described in the Materials and methods section.

The kinetic parameters of wild-type and mutant enzymes in the presence of a synthetic substrate, *N*-suc-AAPF-pNA, at 40°C are shown in Table 3. The *K*_m_ values of the mutants were similar to that of the wild-type enzyme, except that the *K*_m_ value of D212N was slightly higher than that of the wild-type enzyme and the other mutants. The *k*_cat_ values of the mutants were in the range of 47.2-110 s^−1^; these values were 52-120% of the wild-type enzyme value. This result suggests that depletion of a salt bridge or introduction of a new salt bridge to these sites does not profoundly affect the integrity of the structure of AQN at 40°C. Structural integrities of mutants were also confirmed below by the similarity between CD spectra of wild type and mutant enzymes (see Figure 4).

**Table 3:** **Kinetic parameters of the wild-type and mutant enzymes**^**1**^

**Enzyme**	***k***_**cat**_**(s**^**−1**^**)**	***K***_**m**_**(mM)**	***k***_**cat**_**/*K***_**m**_**(mM**^**−1**^ **s**^**−1**^**)**
Wild-type	91.6 ± 2.75	0.79 ± 0.04	116
D17N	110 ± 4.48	0.98 ± 0.05	113
D212N	75.2 ± 3.13	1.10 ± 0.10	68.6
E237Q	96.1 ± 0.98	0.91 ± 0.03	105
D58N	68.5 ± 1.37	0.99 ± 0.07	70.0
D138N	59.5 ± 6.14	0.77 ± 0.01	77.3
D183N	53.0 ± 3.03	0.74 ± 0.05	71.6
G61D	47.2 ± 1.77	0.82 ± 0.13	63.6
G262D	61.3 ± 1.86	0.93 ± 0.15	66.2
S277D	73.6 ± 5.51	0.80 ± 0.12	92.5

^1^Parameters were determined at 40°C with *N*-suc-AAPF-pNA as a substrate. The experiments were performed in either duplicate or triplicate.

### Unfolding study on AQN proteins based on CD measurements

To analyze the unfolding process of active AQN mutants at a fixed temperature, changes in the CD spectra of the wild-type enzyme, D17N, E237Q, D212N or D183N as a function of time were recorded at 70°C and 80°C. Figure 4a and b show the CD spectra of the wild-type enzyme during the 30-min incubations at 70°C and 80°C, respectively. The spectrum was not significantly changed during the 30-min incubation at 70°C. However, at 80°C, the ellipticity at 222 nm was slightly but significantly decreased in the first 5 min, and it subsequently remained unchanged for up to 30 min. Figure 4c and d show the CD profiles of D17N during the 30-min incubations at 70°C and 80°C, respectively. The change in ellipticity at 222 nm at 70°C followed a time course that was very similar to that of the wild-type enzyme at 80°C. However, at 80°C, the initial change at 5 min was slightly more pronounced and there was a gradual decrease in ellipticity that continued for up to 30 min in the D17N mutant. The change in the CD spectrum of E237Q was similar to that of D17N during incubation at both 70°C and 80°C (Figure 4e and f). These results indicated that the secondary structure contents of D17N and E237Q did not show major decreases for 30 min at 70°C. The change in the CD profile of D212N was very similar to that of D17N at 70°C (Figure 4g). However, at 80°C, the ellipticity of D212N in the 200–240 nm range continued to decrease for up to 30 min in parallel with the rapid inactivation of this mutant at 80°C (Figures 4h and 2b). The change in the CD profile of D183N at 70°C is shown in Figure 4i. The peak at 222 nm gradually decreased as a function of time down to 40% of the value before treatment, indicating that more than half of the secondary structures of D183N were destroyed. A comparable change in the ellipticity of D212N occurred only at 80°C. These results suggest that a salt bridge involving Asp183 plays a significant role in maintaining the structure of AQN. This may also be true of other members of the proteinase K subfamily, as the salt bridge involving Asp183 is conserved among these enzymes.

The denaturation curve of the PMSF-treated D183N mutant as monitored by the change in the ellipticity at 220 nm is shown in Additional file1: Figure S1. The *T*_m_ value of D183N was estimated to be 74°C. This is consistent with the extensive decrease of 222 nm peak intensity observed in Figure 4i. Denaturation of other mutants as well as the wild type enzyme apparently occurred at much higher temperature range than D183N mutant, but we could not obtain reliable estimates for their *T*_m_ values. It should be noted that the change in CD spectrum gradually proceeded over 30 min at constant temperature as shown in Figure 4, while measurement of *T*_m_ was carried out at a heating rate of 1°C/min. It is possible that the rate of protein unfolding did not catch up with the elevation of the temperature, and this might give apparent *T*_m_ higher than true *T*_m_.

## Discussion

Subtilases, a group of serine proteases in the subtilisin superfamily, are composed of approximately 275 amino acid residues. Mutation studies on more than 50% of the amino acid residues in their primary structure have been described in the literature (Bryan [2000]). Various factors appear to make complex contributions to subtilase stability. There are six subfamilies in the subtilisin superfamily, and it is possible that the mechanism by which thermal stability is conferred may differ from one subfamily to another. In this report, we investigated the roles of salt bridges in the thermal stabilization of AQN, a proteinase K subfamily member.

Regarding the salt bridges intrinsic to AQN (Asp17-Arg259, Arg31-Glu237 and Arg43-Asp212), the *k*_cat_ values of D17N and E237Q at 40°C and the activities at elevated temperatures toward a synthetic substrate were slightly increased compared to that of the wild-type enzyme; however, these mutants were apparently less thermostable than the wild-type enzyme during heat treatment experiments. These results suggest that the decline of the residual activities of the mutant enzymes during prolonged incubation at high temperatures might be caused in part by extensive autolysis under conditions in which these enzymes exhibit higher activity than the wild-type enzyme. However, it should also be noted that examination by CD spectrometry indicated that D17N and E237Q showed a small change in ellipticity at 222 nm in the first 5 min at 70°C, although the ellipticity remained unchanged over the course of the subsequent incubation for up to 30 min (Figure 4c and e). These results suggest that incubation at 70°C may affect regions of the D17N and E237Q mutants that are not rich in secondary structures, including loop regions, but that the structural perturbation due to the D17N or E237Q mutation may destabilize the active site conformation during a prolonged incubation at 70°C and above. This result agrees with a previous report indicating that the D17N mutant exhibited reduced thermal stability compared to the wild-type enzyme (Arnórsdóttir et al. [2011]).

Recently, Jakob et al. reported an intensive analysis of the roles of charged amino acid residues in a *Bacillus gibsonii* subtilisin protease, BgAP, using site-directed mutagenesis. BgAP Q230E showed increased thermal resistance compared to wild-type BgAP (Jakob et al. [2013]). This result is consistent with our data on E237Q. Glu237 of AQN corresponds to Gln230 of BgAP, and disruption of a salt bridge in E237Q resulted in a rapid decrease of activity during incubation at 70°C and 80°C.

The stability of D212N was similar to that of the wild-type enzyme at 70°C; however, it was inactivated rapidly at 80°C (Figure 2a and b). This result is consistent with the results of the CD spectrometry analysis showing that the secondary structure content was rapidly decreased as a function of time at 80°C. The inactivation mechanism of D212N at 80°C may be different from that of D17N and E237Q at 70°C.

Introducing additional salt bridges at positions mimicking VPR-intrinsic salt bridges in G61D, G262D and S277D resulted in *k*_cat_ values lower than that of the wild-type enzyme; however, this did not affect the thermal stability.

**Figure 5:**
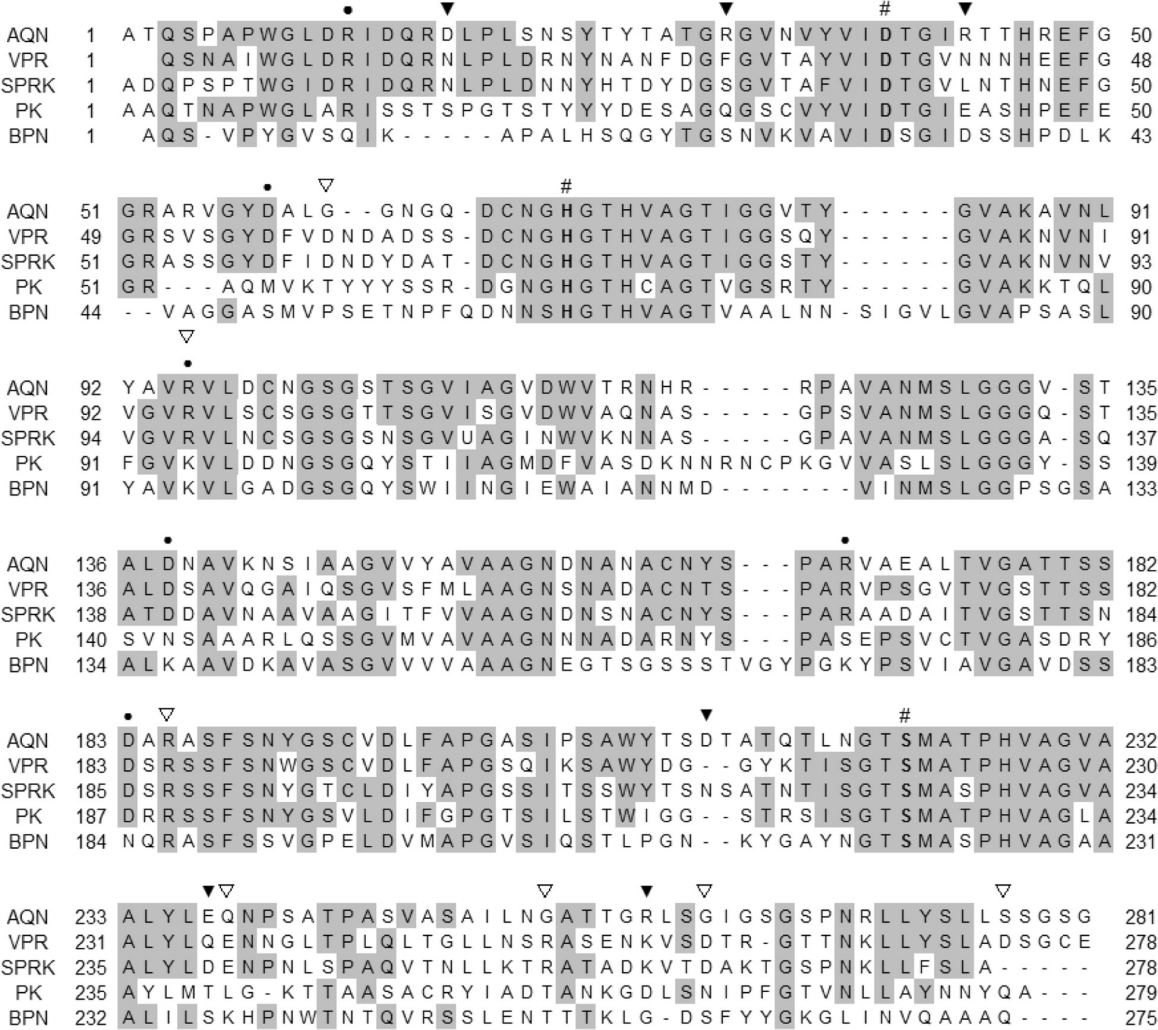
**Alignment of the primary structures of proteinase K subfamily enzymes and subtilisin BPN’.** AQN, aqualysin I; VPR, protease from *Vibrio* sp. PA-44; SPRK, proteinase K-like enzyme from *Serratia* sp.; PK, proteinase K from *Tritirachium album* Limber; BPN, subtilisin BPN’ from *Bacillus amyloliquefaciens*. “#” denotes the catalytic residues Asp, His and Ser. The positions of common (closed circles), AQN-intrinsic (open arrowheads) and VPR-intrinsic (closed arrowheads) salt bridges are represented.

D183N, in which one of the three salt bridges common to AQN and VPN was destroyed, was the most heat-labile among all the mutants tested in this study. Asp183 is conserved among the proteinase K subfamily members and is capable of forming a salt bridge toward an Arg residue. The Asp residue is not conserved among other subtilases (e.g., subtilisin BPN’) (Figure 5). Monitoring of the unfolding process revealed that the secondary structure content decreased as a function of time at 70°C (Figure 4i). The temperature-activity profile shown in Figure 3b indicates that D183N retained full activity during a brief activity measurement period; however, during prolonged incubation at 70°C, the mutant was progressively denatured.

**Figure 6:**
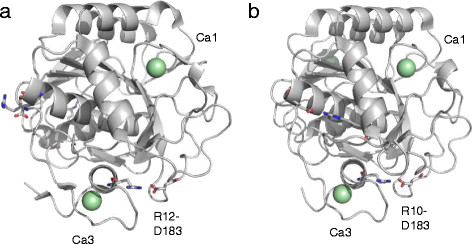
**Crystal structures of AQN (a, 4DZT) and VPR (b, 1SH7).** The positions of the salt bridges, **(a)** Arg12-Asp183 of AQN and **(b)** Arg10-Asp183 of VPR, are represented as stick models. Calcium ions (Ca1 and Ca3) are represented as pale green spheres.

**Figure 7:**
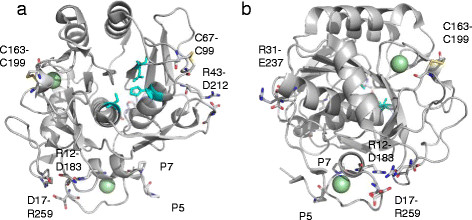
**Crystal structure of AQN (4DZT) with residues contributing to enzyme stabilization. (a)** The residues contributing to the salt bridges Arg12-Asp183, Asp17-Arg259, Arg31-Glu237 and Arg43-Asp212 and the disulfide bridges Cys67-Cys99 and Cys163-Cys199 are indicated and are represented as stick models. Calcium ions are represented as pale green spheres. The catalytic residues are represented as blue stick models. **(b)** The structure is rotated approximately 90 degrees horizontally from **(a)**.

Arg12-Asp183 may serve as a link between the N-terminal α-helix and the long loop region within the 6th and 7th beta-sheets (as named by Siezen and Leunissen (Siezen & Leunissen [1997])) and is located near the Ca3 site (Figure 6a and b). The gradual decrease in the secondary structure content of D183N at 70°C observed by CD spectrometry is most likely initiated by the detachment of these regions. In our previous studies, we demonstrated that Pro residues in the N-terminal loop structure contribute greatly to the thermal stability of AQN and that the disulfide bond, Cys163-Cys194, is important for conformational stability (Sakaguchi et al. [2007]; Sakaguchi et al. [2008b]). The present results on D183N also emphasize the importance of the N-terminal loop region as well as the region including the Cys163-Cys194 disulfide bond and Asp183. The Asp17-Arg259 salt bridge may also be contributing to the stabilization of AQN to a lesser extent because Asp17 is also positioned near the N-terminal region. In addition, the Arg31-Glu237 and Arg43-Asp212 salt bridges appear to serve as additional stabilizing factors that are unique to AQN (Figure 7a and b).

In conclusion, mutation of a salt bridge-forming amino acid that is highly conserved among members of the proteinase K subfamily, Asp183 of AQN, destabilized the protein structure, indicating that this salt bridge plays the most important role in the stability of AQN compared with the other salt bridges. Furthermore, AQN-intrinsic salt bridges confer additional thermal stability to AQN.

## Competing interests

The authors declare that the research was conducted in the absence of any commercial or financial relationships that could be construed as potential conflicts of interest.

## Authors’ contributions

MS conceived of the study, participated in its design and coordination, performed the experiments, interpreted the data and drafted the manuscript. KO participated in its design and coordination, performed the experiments, interpreted the dates and helped to draft the manuscript. SM performed the experiments and the data analysis. NO performed the experiments and the data analysis. YS conceived of the study, participated in its design and coordination, and interpreted the data. FO conceived of the study, participated in its design and coordination, interpreted the data and helped to draft the manuscript. MK conceived of the study, participated in its design and coordination, interpreted the data and drafted the manuscript. All authors read and approved the final manuscript.

## Additional file

## Supplementary Material

Additional file 1: Figure S1. Normalized denaturation curve of PMSF-treated D183N. The denaturation curve is normalized according to Fu = (y_ƒ_-y)/(y_ƒ_-y_u_), assuming a two-state transition, where y_ƒ_ and y_u_ are the CD signals at 220 nm for the folded and unfolded states, respectively, and y is the CD signal at 220 nm at each data point. Click here for file
