# Supplementary figures and images for: Highly conserved salt bridge stabilizes a proteinase K subfamily enzyme, Aqualysin I, from Thermus aquaticus YT-1
Source: AMB Express. 2014 Aug 13;4:59. doi: 10.1186/s13568-014-0059-2 (PMC4131155; doi:10.1186/s13568-014-0059-2)

## Slide 1
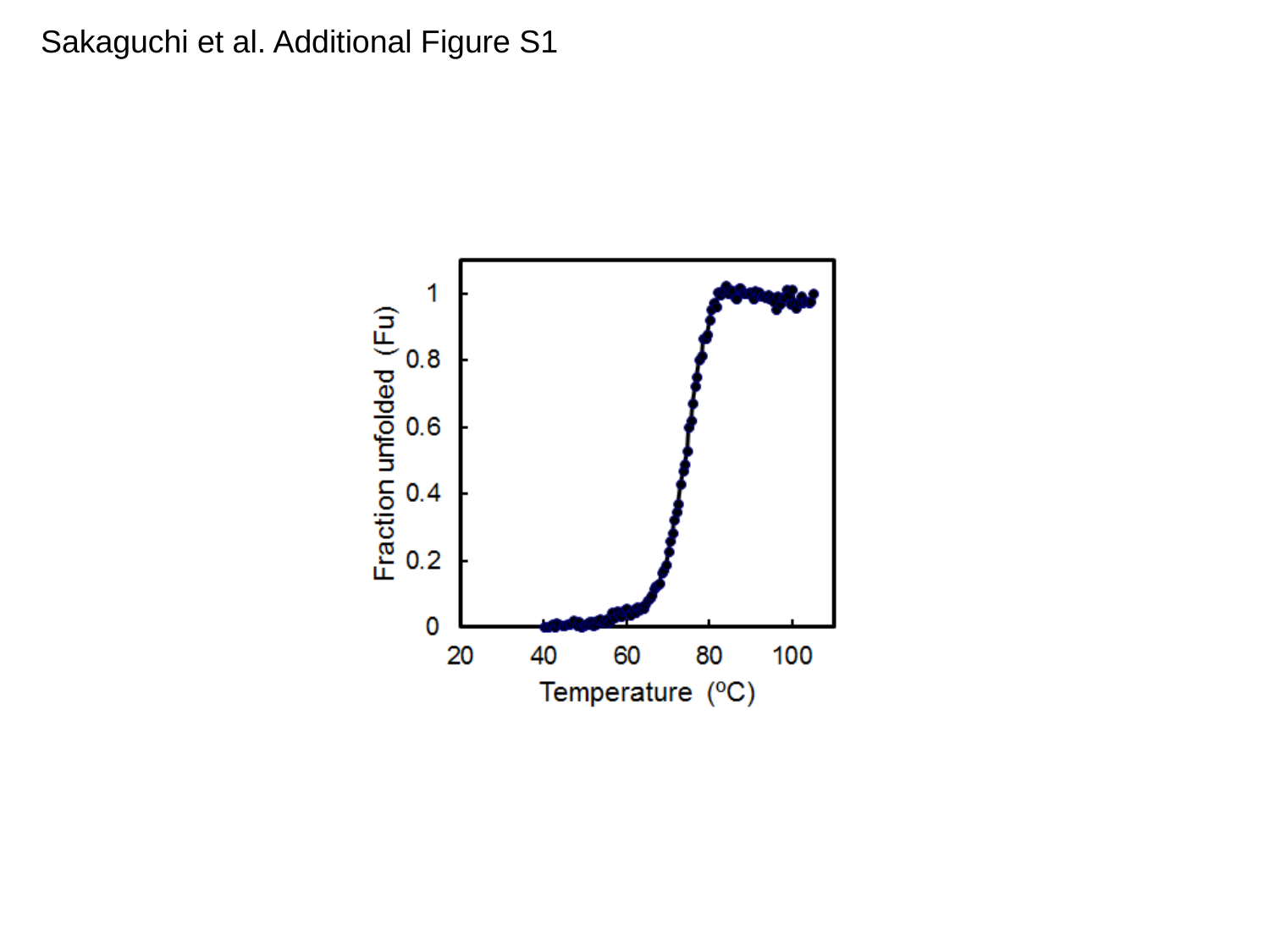

Sakaguchi et al. Additional Figure S1

Supplement: Additional file 1: Figure S1. — Normalized denaturation curve of PMSF-treated D183N. The denaturation curve is normalized according to Fu = (yƒ-y)/(yƒ-yu), assuming a two-state transition, where yƒ and yu are the CD signals at 220 nm for the folded and unfolded states, respectively, and y is the CD signal at 220 nm at each data point. [file s13568-014-0059-2-S1.pptx]
